# Maternal gut *Bifidobacterium breve* modifies fetal brain metabolism in germ-free mice

**DOI:** 10.1016/j.molmet.2024.102004

**Published:** 2024-08-08

**Authors:** Jorge Lopez-Tello, Raymond Kiu, Zoe Schofield, Cindy X.W. Zhang, Douwe van Sinderen, Gwénaëlle Le Gall, Lindsay J. Hall, Amanda N. Sferruzzi-Perri

**Affiliations:** 1 Department of Physiology, Development, and Neuroscience, Centre for Trophoblast Research, University of Cambridge, Cambridge, UK; 2 Department of Physiology, Faculty of Medicine. Autonomous University of Madrid, Spain; 3 Food, Microbiome & Health, Quadram Institute Bioscience, Norwich Research Park, Norwich, UK; 4 APC Microbiome Institute, University College Cork, Cork, Ireland; 5 Norwich Medical School, University of East Anglia, Bob Champion Research and Education Building, James Watson Road, Norwich Research Park, Norwich NR4 7UQ, UK; 6 Institute of Microbiology & Infection, University of Birmingham, Birmingham, UK; 7 Department of Microbes, Infection & Microbiomes, School of Infection, Inflammation & Immunology, University of Birmingham, Birmingham, UK

**Keywords:** Pregnancy, Microbiota, Fetus, Brain, Metabolism

## Abstract

**Background:**

Recent advances have significantly expanded our understanding of the gut microbiome's influence on host physiology and metabolism. However, the specific role of certain microorganisms in gestational health and fetal development remains underexplored.

**Objective:**

This study investigates the impact of *Bifidobacterium breve* UCC2003 on fetal brain metabolism when colonized in the maternal gut during pregnancy.

**Methods:**

Germ-free pregnant mice were colonized with or without *B. breve* UCC2003 during pregnancy. The metabolic profiles of fetal brains were analyzed, focusing on the presence of key metabolites and the expression of critical metabolic and cellular pathways.

**Results:**

Maternal colonization with *B. breve* resulted in significant metabolic changes in the fetal brain. Specifically, ten metabolites, including citrate, 3-hydroxyisobutyrate, and carnitine, were reduced in the fetal brain. These alterations were accompanied by increased abundance of transporters involved in glucose and branched-chain amino acid uptake. Furthermore, supplementation with this bacterium was associated with elevated expression of critical metabolic pathways such as PI3K-AKT, AMPK, STAT5, and Wnt-β-catenin signaling, including its receptor Frizzled-7. Additionally, there was stabilization of HIF-2 protein and modifications in genes and proteins related to cellular growth, axogenesis, and mitochondrial function.

**Conclusions:**

The presence of maternal *B. breve* during pregnancy plays a crucial role in modulating fetal brain metabolism and growth. These findings suggest that *Bifidobacterium* could modify fetal brain development, potentially offering new avenues for enhancing gestational health and fetal development through microbiota-targeted interventions.

## Highlights

• *B. breve* in maternal gut alters fetal brain cell cycle and axogenesis genes.
• *B. breve* in maternal gut alters fetal brain metabolism, reducing 10 key metabolites.
• *B. breve* in maternal gut modifies nutrient transporters in fetal brain.
• *B. breve* impacts mitochondrial TCA cycle, HIF and Wnt-β-catenin in fetal brain.

## 1 Introduction

Fetal growth restriction (FGR) is a severe condition defined as the failure of the fetus to reach its growth potential due to pathological compromise. A main cause of FGR is placental insufficiency during gestation [1]. Human epidemiological studies and experimental animal models have found that FGR and placental insufficiency can affect fetal organ development, including changes in key organs like the heart, kidneys and brain [[2], [3], [4], [5], [6], [7], [8], [9]]. Studies have also shown that disruption of fetal brain development due to suboptimal intrauterine environments can lead to neurodevelopmental disorders postnatally, including motor and cognitive dysfunctions, learning impairments and cerebral palsy [[10], [11], [12], [13], [14], [15]]. Managing placental insufficiency and FGR in a clinical setting can present substantial challenges. Pharmacological interventions like aspirin, heparin and sildenafil citrate, which target inflammatory, coagulation and blood flow pathways are controversial due to variability in their effects on feto-placental and pregnancy outcomes [16,17]. Hence, there is an imminent need to devise effective treatments that can prevent and/or mitigate the adverse consequences associated with FGR.

In recent years, there has been an explosion of studies describing the importance of the gut microbiota in regulating developmental processes, from neurogenesis [18] to ageing [19,20]. Moreover, a perturbed gut microbiota has been linked to neurological conditions, like Parkinson's disease [21] and schizophrenia [22], and metabolic-related disorders, including type-2 diabetes [23]. In the context of pregnancy, previous studies have demonstrated dramatic changes in the composition of the maternal gut microbiota during pregnancy [24] and in women who developed a hypertensive disorder of pregnancy, preeclampsia or had abnormal placental growth [25,26]. Of note, the genus *Bifidobacterium* increases in abundance in the maternal gut during pregnancy in both women and mice [27] and has been shown to possess multiple benefits. For example, *Bifidobacterium* protects against infectious diseases and is involved in modulating host immune responses [[28], [29], [30], [31]]. Moreover, we have previously shown that administering three consecutive doses of *Bifidobacterium* (specifically, *Bifidobacterium breve* UCC2003) on gestational days 10, 12, and 14 to pregnant germ-free (GF) mice (referred to as the BIF group throughout the text; see Fig. S1), and non-pregnant specific-pathogen-free mice results in stable gut colonization [29,32]. This timing and dosing were chosen based on the observation that levels of *Bifidobacterium* increase throughout pregnancy [27] and to reduce the need for repeated handling of the mice, which can lead to stress and spontaneous abortions. Additionally, our approach could potentially align with supplementation studies in pregnant women, as we started the experiments after pregnancy was confirmed.

Herein we tested the hypothesis that maternal gut *B. breve* abundance would associate with changes in the development and metabolism of the fetal brain. Working with germ-free mice that lack a maternal microbiota (GF) and GF mice that received *B. breve* UCC2003 treatment during pregnancy, our results demonstrate the significance of maternal gut *B. breve* in fetal brain development and metabolism.

## 2 Results

### 2.1 Maternal gut *B. breve* UCC2003 modifies expression of genes involved in cell cycle and axogenesis

Using this approach, we have previously shown that administration of *B. breve* UCC2003 to pregnant GF mice improves fetal growth together with beneficial structural and functional alterations in the placenta and changes in metabolic genes in the fetal liver [32]. Moreover, supplementation with *B. breve* UCC2003 (BIF) improved liver size compared to untreated GF mice (GF). However, brain weight did not differ between untreated GF and BIF fetuses, suggesting that although untreated GF fetuses were growth restricted, they exhibited preserved brain development [32]. To understand the molecular basis of this, we used biobanked samples from our previous study and quantified the mRNA levels of key growth, cell cycle, microglia, and neurogenesis genes in the brain of fetuses from GF mice following maternal *B. breve* UCC2003 administration. This revealed that there was no difference in the expression of vascular gene *Vegf* or apoptotic genes *Tp53*, *Casp3* and *Bax* in the brain between fetuses of the GF and BIF groups (Figure 1A). However, mRNA levels of the transcriptional activator *Foxm1* and the mitotic cycling *Cdk1* gene were significantly reduced in the fetal brain of the BIF compared to the untreated GF group (Figure 1B). No changes were observed in other assessed cell cycle genes (*Cdk2*, *Cdk4, Cdc42, Ccne1, Gli1 and Axin2*) or in genes involved in microglia activation (e.g. *Ier3*, *Klf2* or *Egr1)* between GF and BIF groups (Figure 1B–C). Quantification of expression levels of key axonogenesis genes revealed that *Plxna3* was significantly down-regulated in the fetal brain of BIF compared to untreated GF mice (Figure 1D). Also, *Slit1* showed a tendency to be reduced in the BIF treated group (p = 0.05). The expression levels of the other axonogenesis genes assessed (*Sema3f*, *Ntn1*, *Nrcam, Sv2b, Gabra1, Gabrg1 and Gabrg2*) were shown to be similar between the two experimental groups (Figure 1D). Collectively, these data demonstrate that maternal gut *B. breve* UCC2003 modulates the expression of specific genes involved in cell cycle control and axonogenesis in the fetal brain.

**Figure 1:**
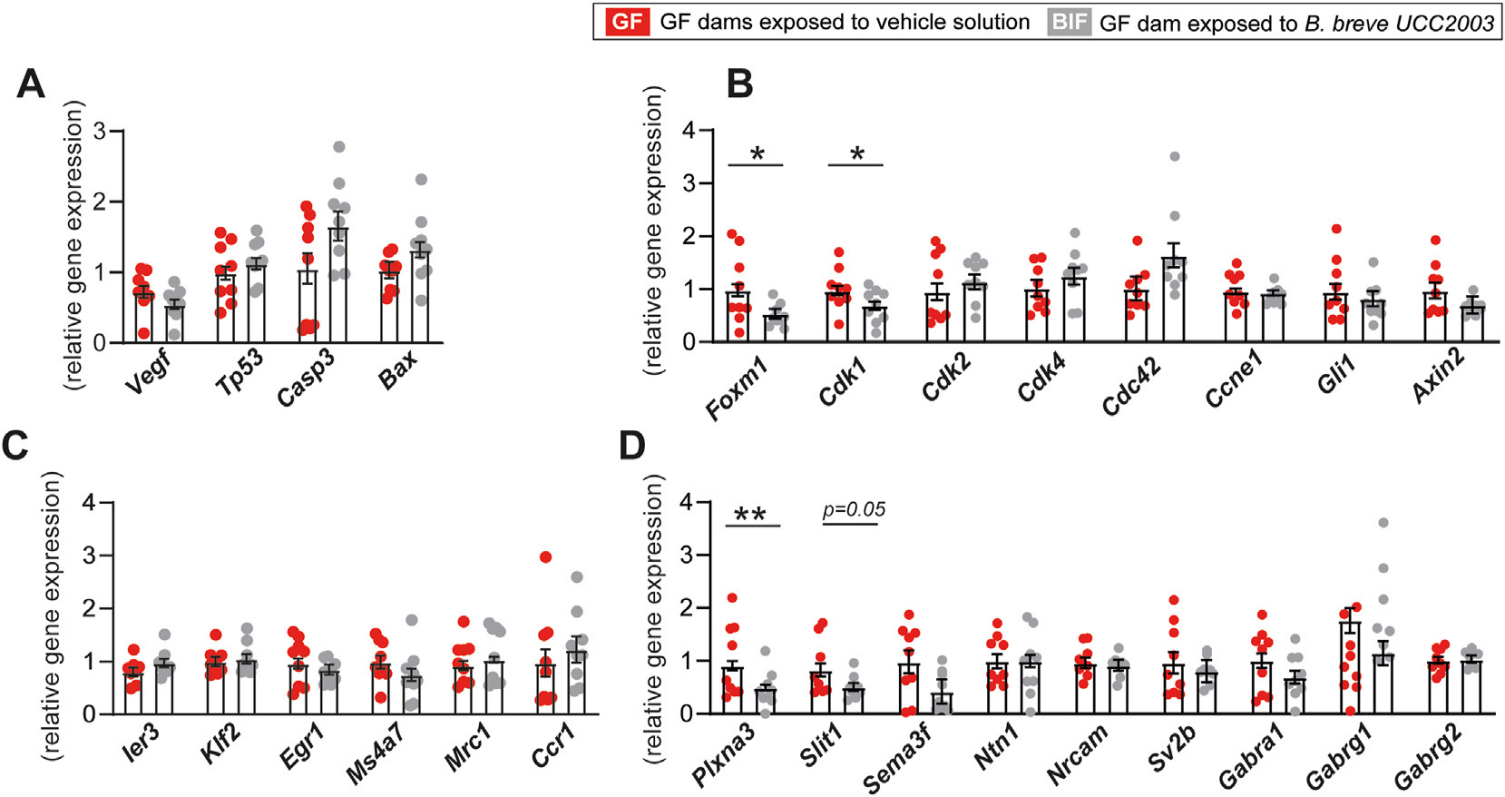
**Maternal gut *B. breve* supplementation modifies genes involved in fetal brain cell growth and axon development.** Relative mRNA levels of genes involved in cell cycle (A-B), microglia activation (C), and axonogenesis (D). Gene expression is relative to two housekeeping genes (*Gapdh* and *Actb*). Data analysed by ANOVA with the group as fixed effect and means comparisons was made by Fisher test (further details in the methods section). Data are means ± SEM with individual data points shown. ∗*P* < 0.05; ∗∗*P* < 0.01.

### 2.2 Maternal gut *B. breve* UCC2003 modifies fetal brain metabolism

**Figure 2:**
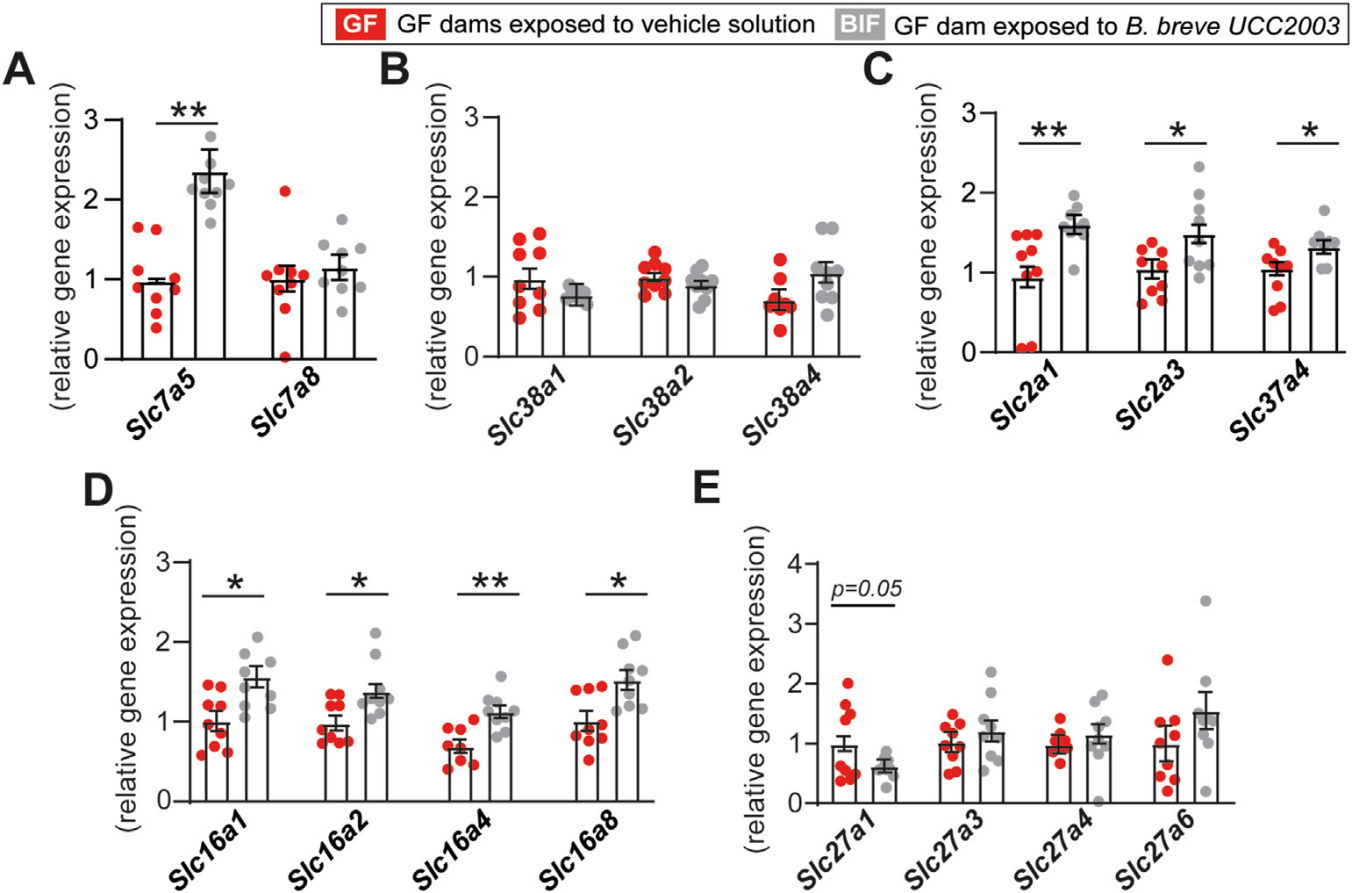
**Maternal gut *B. breve* supplementation modifies the expression of nutrient transporters in the fetal brain.** (A–E) Relative mRNA levels of genes encoding nutrient transporters measured in fetal brain samples, expression is relative to two housekeeping genes (*Gapdh* and *Actb*). Statistical analysis performed by ANOVA with the group as fixed effect (each fetus as a repeated measure) and means comparisons was made by Fisher test (linear mixed model). Data are means ± SEM with individual datapoints shown. Data obtained from a total of 5 GF and 6 BIF pregnant dams/litters (9 fetuses per group analysed). ∗*P* < 0.05; ∗∗*P* < 0.01.

To investigate if maternal *B. breve* UCC2003 supplementation affects fetal brain metabolism, we performed metabolomic profiling of fetal brain lysates from untreated and BIF treated GF mice. We quantified a total of 78 metabolites, of which 10, primarily amino acids and citrate were significantly reduced in the BIF compared to untreated GF group (Table 1 and Table S1). Considering that the levels of certain amino acids such as leucine and valine were altered in the BIF group, we quantified the mRNA levels of transporters involved in branched-chain amino acid uptake and metabolism in the fetal brain. This analysis identified that expression of the large neutral amino acid transporter 1 (LAT1), encoded by the *Slc7a5* gene, but not large neutral amino acid transporter 2 (LAT2; encoded by *Slc7a8*) was significantly elevated in the BIF treated group compared to untreated GF mice (Figure 2A). No differences were found in the expression levels of genes encoding other amino acid transporters, namely the system A amino acid transporters (*Slc38a1,2,4 - SNATs*) in the fetal brain between BIF versus untreated GF mice (Figure 2B).

**Table 1:** **Maternal gut *B. breve* supplementation induce changes in fetal brain metabolites.** Data analysed by one-way ANOVA, with the group as fixed effect and means comparisons made by Fisher test (general linear model-GLM model). Litter size added as a covariate. Data displayed as mean ± SEM. Values were considered statistically significant with P < 0.05. Further data about the metabolites analysed can be found in Table S1.

Metabolite (mmol/Kg)	GF group (n = 5)	BIF group (n = 5)	P value
3-Hydroxyisobutyrate	0.063 ± 0.004	0.041 ± 0.007	0.048
Alanine	3.523 ± 0.578	1.776 ± 0.163	0.020
Arginine	0.192 ± 0.030	0.107 ± 0.015	0.045
Aspartate	1.937 ± 0.228	1.119 ± 0.150	0.026
Carnitine	0.370 ± 0.047	0.210 ± 0.023	0.022
Dimethylamine	0.481 ± 0.192	0.072 ± 0.053	0.002
Leucine	0.261 ± 0.019	0.177 ± 0.017	0.027
Threonine	2.209 ± 0.292	1.170 ± 0.249	0.020
Valine	0.378 ± 0.048	0.262 ± 0.027	0.035
Citrate	0.219 ± 0.025	0.154 ± 0.023	0.046

In previous work we reported that circulating levels of glucose were increased in response to *B. breve* UCC2003 administration of GF pregnant mice [32]. Glucose is the predominant source of energy used by the fetal brain to support its development [33]. Hence, we quantified the mRNA levels of the main glucose transporter-encoding genes *Slc2a1* and *Slc2a3* (GLUT1 and GLUT3, respectively) in fetal brain tissue. This revealed that expression of both glucose transporters was significantly elevated in the fetal brain of BIF treated mice compared to untreated GF group (Figure 2C). Moreover, the mRNA level of *Slc37a4* (encoding G6PT), an important gene that controls glucose homeostasis by regulating the transport of glucose-6-phosphate from the cytoplasm to the lumen of the endoplasmic reticulum [34], was also elevated in the BIF group (Figure 2C). In addition, the expression of solute carrier family 16 members 1,2,4,8 - MCTs which are involved in the movement of monocarboxylates, including lactate and pyruvate, and ketone bodies like d-β-hydroxybutyrate [35], were all shown to be significantly elevated in the fetal brain of BIF treated pregnant mice compared to the untreated GF group (Figure 2D). There was no difference in the expression of genes involved in lipid uptake (solute carrier family 27 members - FATP) in brain lysates of BIF treated pregnant GF mice compared to untreated GF mice (Figure 2E). These findings indicate that the presence of maternal gut *B. breve* UCC2003 impacts on the levels of specific metabolites and expression of key nutrient transporters in the fetal brain.

### 2.3 Maternal gut *B. breve* UCC2003 modifies cellular and metabolic pathways in the fetal brain

Driven by the changes detected in metabolites and nutrient transporters, we proceeded to analyze cellular and metabolic pathways in the fetal brain. Therefore, we analysed a variety of signalling pathways that have been previously implicated in the execution of different processes in the brain, including: cellular growth, proliferation and survival (e.g. PI3K-AKT [36,37]), nutrient uptake (e.g. AMPK [38]), dendritic organization (e.g. ERK [39]), neuronal development/differentiation and astrogliogenesis (e.g. AMPK [40], STAT3-5 [41]), and cell fate transition (e.g. Wnt/β-catenin [42]).

**Figure 3:**
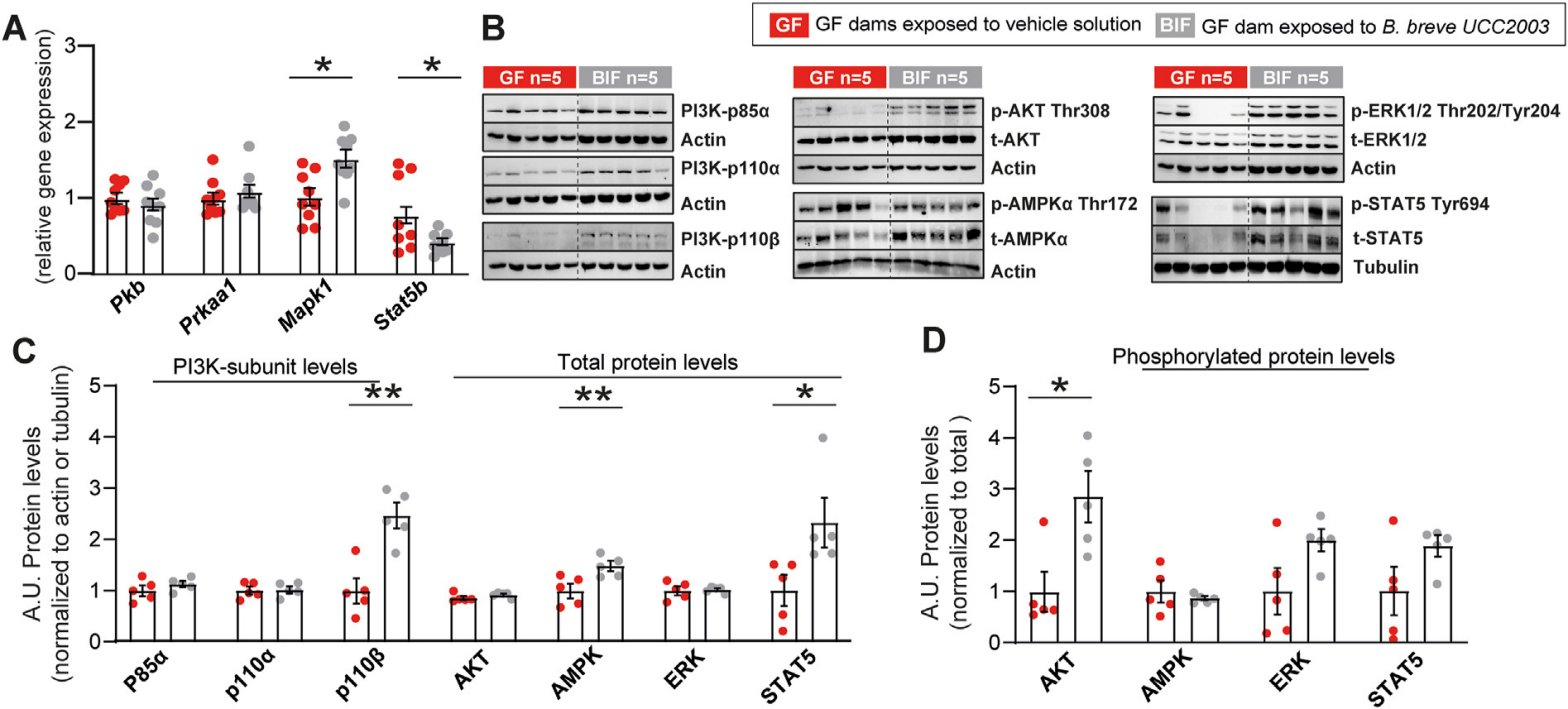
**Maternal gut *B. breve* supplementation modifies genes and proteins involved in fetal brain cell metabolism and growth.** (A) Relative mRNA levels of genes encoding cell signalling pathways measured in fetal brain samples, expression is relative to two housekeeping genes (*Gapdh* and *Actb*). (B–F) Immunoblots and relative protein expression values of proteins involved in cellular metabolism and growth. Protein levels were normalized to total protein (in the case of phosphorylated proteins), actin or tubulin abundance. Data analysed by ANOVA with the group as fixed effect and means comparisons was made by Fisher test (further details in the methods section). Data are means ± SEM with individual datapoints shown. ∗*P* < 0.05; ∗∗*P* < 0.01.

Between the BIF and untreated GF groups, at gene level, we observed elevated levels of *Mapk1* (encoding mitogen-activated protein kinase 1), reduced *Stat5b* (signal transducer and activator of transcription 5B), and unchanged expression of *Pkb* and *Prkaa1* (encoding AKT serine/threonine kinase 1 and protein kinase AMP-activated catalytic subunit alpha 1, respectively) in the fetal brain (Figure 3A). Immunoblotting analysis of PI3K-AKT, MAPK, AMPK and STAT5 signalling pathways (Figure 3B) however revealed increased levels of PI3K-p110β, AMPK and STAT5 proteins in fetal brain lysates of BIF treated compared to untreated pregnant GF mice (Figure 3C). Informed by phosphorylation levels, we also observed enhanced activation of the AKT signalling pathway, indicated by increased threonine 308 residue phosphorylation levels. No differences were observed in the other signalling pathways (Figure 3D). Collectively, these data show that the presence of maternal gut *B. breve* UCC2003 is associated with the activation of key cellular and metabolic pathways in the fetal brain.

### 2.4 Maternal gut *B. breve* UCC2003 induces changes in HIF affecting mitochondrial TCA cycle and Wnt-β-catenin signalling in the fetal brain

**Figure 4:**
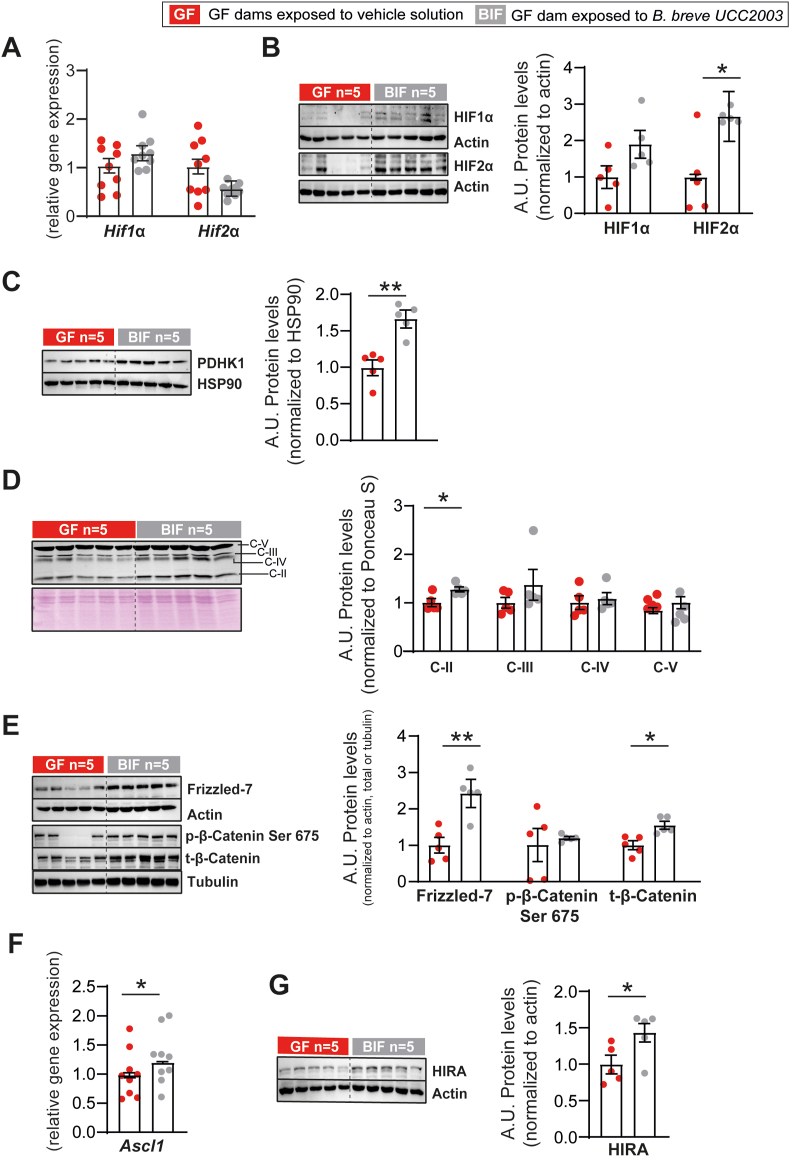
**Maternal gut *B. breve* supplementation results in stabilization of HIF2α and changes in mitochondrial function and Wnt-β-catenin signalling in the fetal brain.** (A) Relative mRNA levels of *Hif1α* and *Hif2α.* (B-C) Immunoblots and relative protein expression values of HIF1α, HIF2α and PDHK1 normalized to actin or HSP90 levels. (D) Immunoblot and relative protein abundance of mitochondrial complexes (C) normalized to Ponceau Staining. (E) Immunoblots and relative protein abundance of Frizzled-7 and β-catenin normalized to actin or tubulin levels. (F) Relative mRNA levels of *Ascl1*. (G) Immunoblot and relative protein abundance of HIRA. All gene expression is relative to two housekeeping genes (*Gapdh* and *Actb*). Western blotting performed with 5 fetuses/group from 5 dams per group (different litters). Data analysed by ANOVA with the group as fixed effect and means comparisons was made by Fisher test (further details in the methods section). Data are means ± SEM with individual datapoints shown. ∗*P* < 0.05; ∗∗*P* < 0.01.

Molecular pathways like the PI3K-AKT can regulate the stability of hypoxia inducible factors (HIFs) [43]. Moreover HIFs regulate brain development and function even in normoxic conditions [44]. We therefore analysed the mRNA and protein levels of HIF-1α and HIF-2α. Whilst we found no difference in the mRNA levels of *Hif1α* or *Hif2α* (Figure 4A), HIF-2α protein abundance was significantly up-regulated in the fetal brain of BIF treated pregnant mice compared to the untreated GF group (no change HIF-1α; Figure 4B). HIF can regulate the tricarboxylic acid (TCA) cycle by repressing mitochondrial function and activating expression of the gene encoding pyruvate dehydrogenase kinase 1 (PDHK1) [45]. We therefore measured the abundance of PDHK1 in fetal brain lysates and found that total protein content was significantly elevated in the BIF treated group compared to the GF (Figure 4C). Moreover, we quantified mitochondrial ATP production capacity by assessing oxidative phosphorylation (OXPHOS) via analysis of OXPHOS complexes. We were able to detect 4 out of the 5 respiratory chain complexes and found that complex-II was significantly elevated in the fetal brain of BIF treated compared to the untreated GF mice. No changes were observed in the levels of other OXPHOS complexes detected (Figure 4D).

HIF is also known to activate multiple genes and signalling cascades, including the Wnt and β-catenin pathway [46,47]. We therefore quantified the levels of proteins in these pathways and observed that the abundance of Frizzled-7, a cognate Wnt receptor [48] and total levels of β-catenin protein were significantly elevated in the fetal brain of BIF treated GF mice (Figure 4E). However, the phosphorylated levels of β-catenin at the serine 675 residue, which promotes β-catenin stability and increases β-catenin transcriptional activity [[49], [50], [51], [52]], were unaltered when normalized to total β-catenin (Figure 4E). Moreover, the abundance of *Ascl1*, a pro-neural transcription factor that also induces the Wnt signalling pathway [53,54] was significantly increased in the brain of fetuses from BIF treated GF mice compared to the untreated GF group (Figure 4F). Finally, the abundance of histone cell cycle regulator (HIRA), a histone chaperone that regulates neurogenesis by enhancing β-catenin expression [55], was enhanced in the fetal brain of GF mice treated with BIF versus untreated GF mice (Figure 4G). Taken together, these results suggest that the presence of *B. breve* in the maternal gut stabilizes HIF2-α, enhances mitochondrial oxidative phosphorylation capacity and promotes accumulation of Wnt and β-catenin levels in the fetal brain.

## 3 Discussion

Previous studies have established that the gut microbiome can influence brain function and behaviour [56,57], including a recent murine model study revealing that the maternal gut microbiota can modulate fetal axonogenesis via microbially regulated metabolites [18]. These data suggest that targeting the maternal gut microbiota, such as through probiotic supplementation, may lead to defined and specific changes in brain development across the perinatal period. Numerous species and strains of *Bifidobacterium* are commonly found in healthy breast-fed infants, as well as being used as probiotics, where they exert a range of beneficial health effects in the host, including protection against inflammatory insults and promoting barrier function and differentiation of intestinal epithelial cells [28,29]. In terms of brain function, prior work has demonstrated that strains of *Bifidobacterium,* like *Bifidobacterium longum* 1714™, can modify brain activity by improving stress responses and cognitive function in human and animal models [[58], [59], [60], [61]]. In the current study, we report that oral administration of *B. breve* UCC2003 to pregnant mice can induce changes in the developing fetal brain, namely through modifying fetal brain metabolism, and genes and signalling pathways involved in cell growth and axonogenesis. Our results have potential translational implications, as around 14% of pregnant women use probiotics during pregnancy in European countries like the Netherlands [62]. However, the utilization of probiotics in clinical and nutritional contexts, particularly during pregnancy, remains a topic of continual debate. Thus, to comprehensively understand their influence on the developing offspring, it is essential to define the mechanisms supporting their beneficial effects.

In this present investigation, our data suggest that *B. breve* UCC2003 strongly influences fetal brain metabolism. Notably, the fetal brain of the BIF group exhibited a significant reduction in ten specific metabolites. These findings are particularly intriguing, as our previous study did not reveal any changes in the concentrations of these metabolites in the fetal liver or in the placental labyrinth zone, thus highlighting that *Bifidobacterium* supplementation induces distinct responses in different fetal tissues [32]. Among the metabolites, multiple amino acids were reduced, suggesting that these amino acids, including alanine, leucine and valine may have been used by the fetus. Moreover, other amino acids like arginine or carnitine have previously been reported to be impacted by the maternal microbiota, as shown in studies comparing fetal brains of GF versus specific pathogen free mice [63]. Leucine, which is transported by the *Slc7a5* (elevated at gene level in the BIF group) and regulated by MTORC1 [64], has been shown to be regulated by fetal glycaemia, as glucose concentration decreases leucine oxidation independent of insulin [65]. Our previous work showed that administration of *B. breve* UCC2003 resulted in upregulation of the *Slc2a1* transporter in the placental labyrinth zone and in the fetal liver. Moreover, fetal glycaemia was improved in the BIF group and compared to the specific-pathogen-free fetus [32]. Although in this study we did not observe changes in the glucose concentrations in the fetal brain, we found that the abundance of key glucose transporters, specifically, *Slc2a1*, *Slc2a3* and *Slc37a1*, were increased in the BIF group. Interestingly, the mRNA levels of *Slc2a3* and *Slc37a1* in the fetal liver were unaltered in the BIF group compared to GF mice [32], highlighting once again the different metabolic responses of the fetus based on tissue type. Future work should explore the role of fetal glycaemia and its impact on the metabolism of amino acids like leucine in the different organs of the fetus.

Another metabolite that was reduced in the BIF group was 3-hydroxybutyrate. This was in line with the lower levels of valine, as 3-hydroxybutyrate serves as a crucial intermediate in the metabolism of branched-chain amino acids like valine [66]. Previous studies indicate that 3-hydroxybutyrate, which can be used as an alternative energy source for the brain and other tissues when glucose becomes scarce [67], is highly involved in mitochondrial function as it can modify mitochondrial membrane permeability transition [68]. Moreover, it is known that neurons treated with 3-hydroxybutyrate exhibit an increase in mitochondrial respiration [68]. We also found two additional metabolites that were reduced in the BIF group, namely carnitine and citrate, which are involved in the tricarboxylic acid (TCA) cycle. Carnitine plays a crucial role in the transport of long-chain fatty acids into the mitochondria [69], whilst citrate is an intermediate of the TCA cycle involved in nicotinamide adenine dinucleotide metabolism [70]. Analysis of mitochondrial electron transfer system (ETS) components revealed that the fetal brains of BIF treated mothers had increased levels of the mitochondrial complex-II. Both citrate and 3-hydroxybutyrate are well known inhibitors of succinate dehydrogenase (mitochondrial complex-II) [71], suggesting a potential link between these results. Moreover, we found that the mRNA levels of the transcription factor *Foxm1* and cyclin *Cdk1* were reduced in the BIF group. Prior work has shown that *Foxm1* and *Cdk1* can also control mitochondrial oxygen consumption rates and mitochondrial abundance [72]. Future experiments could be undertaken to understand the potential interplay between these metabolites and the elevated mitochondrial ETS complex II activity, such as using fetal brain explants or cerebral organoids cultured with and without these metabolites, and using high-resolution mitochondrial respirometry analysis. Moreover, aside from *in vitro* studies to improve our understanding of the mechanisms of action, animal studies using additional control experimental groups are needed [32].

In our study, we did not observe changes in the mRNA levels of well-known genes involved in angiogenesis or apoptosis, such as *Vegf*, *Tp53*, *Casp3* or *Bax*. However, we observed reduced mRNA levels of *Plxna3.* This gene, controlled by the maternal microbiota [18], encodes a class 3 semaphorin receptor that regulates multiple neurodevelopmental processes including axonal growth and guidance [73,74], and neuronal death [75]. Semaphorins have been shown to interact with critical pathways involved in cell proliferation, growth, and apoptosis including the PI3K/AKT pathway [76]. Brains from the BIF group showed activation of the PI3K pathway as evidenced by increased protein levels of PI3K-p110β and elevated phosphorylation levels of AKT. Activation of this signalling pathway has been linked to enhanced dendritic branching, cellular proliferation, neuronal hypertrophy [37]. Moreover, the PI3K/AKT has been implicated in the regulation of MCTs [35,77], which were found to be elevated in the BIF group. In our study it is unclear if the administration of this bacterium induced structural changes in the fetal brain and if so, whether the PI3K/AKT pathway could be driving such changes. Therefore, further experiments using structural/histological analysis are needed in order to determine changes in cell division, cell death, and axon development.

Prior research in mice has demonstrated the crucial role of the HIF-2α in facilitating proper brain formation, neural network development and the migration of neural stem cells [44,78]. Additionally, HIF-2α has been identified as having protective functions for neural stem cells, promoting neurogenesis, and regulating angiogenic and apoptotic processes [79]. In our study, we observed that *B. breve* UCC2003 administration led to the stabilization of HIF-2α in the fetal brain without inducing changes in HIF-1α protein stabilization. The unaltered state of HIF-1α in this model suggests the absence of intrauterine hypoxic conditions in the BIF group. In this regard, previous research has indicated that HIF-2α, but not HIF-1α, is present in the brains of adult mice under normoxic conditions, and that HIF-1α protein stabilization occurs only under hypoxic conditions [44]. However, other authors suggest that the stability of HIF-2α protein is similar to that of HIF-1α and relies on oxygen-dependent degradation [80]. Following this line of research, *in vitro* work using neonatal rat neuronal cultures has demonstrated that hypoxia causes up-regulation of AMPK. This metabolic signalling pathway aside from acting as a metabolic sensor regulating glycolysis, has neuroprotective and pro-apoptotic effects [38]. In our work we found that total levels, but not phosphorylated AMPK, were increased in the BIF group. Moreover, additional pathways that have been associated with HIFs and found dysregulated in this model are the Wnt/β-catenin and STAT5 signalling pathways. Both total protein levels of Wnt/β-catenin and STAT5 were increased and in the case of STAT5, the mRNA levels were decreased, suggesting a potential change in the stability, translational efficiency, and/or protein degradation rates in response to *B. breve* administration [81]. The activity of Wnt/β-catenin pathway is closely related with low oxygen levels [82] and is critical for the development of brain regions, including the proper formation of the midbrain and anterior hindbrain. Inhibition of this pathway results in brain abnormalities due to changes in cell proliferation and cell death [83,84]. STAT5 is a target gene of HIF-2α in haematopoietic stem cells [85] and is particularly important in the forebrain region development and axon guidance [86]. Moreover, we found that PDHK1 was significantly elevated in the BIF group. It is known that HIF can upregulate the expression of PDHK1, which indeed was found elevated in the fetal brain of the BIF group. This adaptive mechanism is used to sustain energy production by inhibiting the conversion of pyruvate to acetyl-CoA, and promoting glycolysis over oxidative phosphorylation [87]. Other studies have found that HIF-2α protein is inhibited or knocked down, the expression of *Ascl1* (which was increased in the BIF group) is enhanced; resulting in the induction of sympathetic nervous system differentiation marker genes [88]. Taken together, our data suggest that oxygen levels may be altered in fetuses exposed to *B. breve* UCC2003 when compared to GF untreated mice. Although we could not measure oxygen levels at the tissue level in this model to verify whether the fetuses were hypoxic or not, we can also speculate another potential mechanism linked to the HIF-2α protein stabilization that may relate to changes in the transport region of the placenta [32]. In this regard, we found that *B. breve* UCC2003 oral administration resulted in a thinner placental barrier thickness, and this reduction would be expected to aid in the diffusion of oxygen from the mother to the fetus [89]. Therefore, it is less likely that the fetuses of BIF pregnant mice are hypoxic, but further work is certainly required.

**Figure 5:**
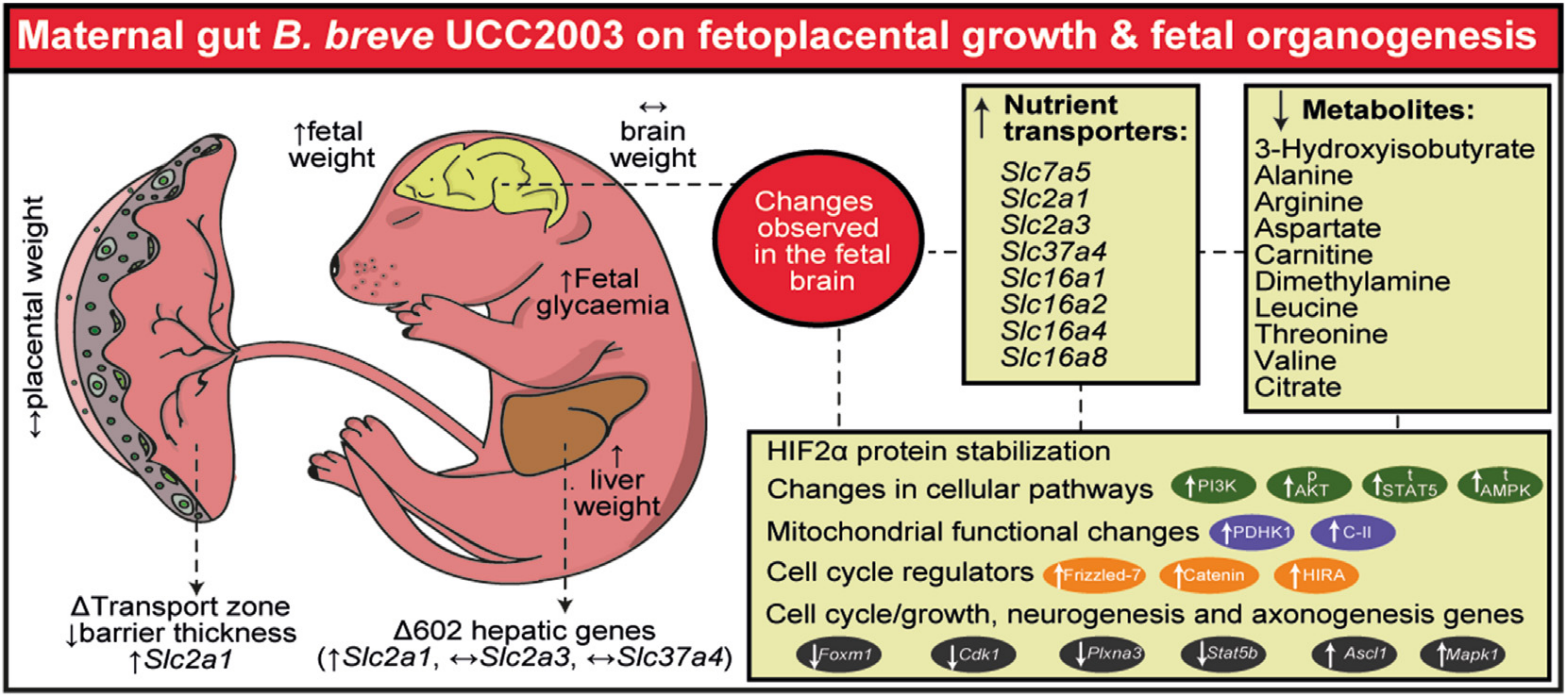
**Summary Figure illustrating changes induced by Maternal gut *B. breve* supplementation.** The illustration summarizes the most relevant results obtained from our previous study [32] and the new study comparing GF versus GF treated with *B. breve* during development. C-II refers to the mitochondrial complex-II OXPHOS. Abbreviations: p (phosphorylated protein levels), t (total protein levels).

While the exact mechanisms linking *B. breve* in the maternal gut and the fetal brain are complex and likely multifaceted, we propose several potential mechanisms. Changes may be initiated by short-chain fatty acids, such as acetate, produced by *B. breve*, as these can affect the vagus nerve, are transported across the placenta and are known to alter blood-brain barrier permeability in both the mother and fetus [[90], [91], [92], [93], [94]]. Another possible mechanism is through the release of bacterial extracellular vesicles (BEVs), which have been linked to changes in host immunity [95]. However, the direct contribution of *B. breve* liberated short-chain fatty acids and BEVs to placental and fetal growth, let alone the developing fetal brain, has yet to be described. As exposed previously [32], our findings suggest that changes in fetal development could be related to alterations in the structure and function of transport labyrinth region of the placenta in *B. breve* supplemented GF mice. However, we have not explored whether *B. breve* could also alter the endocrine region of the placenta, which is a critical determinant of fetal development [96,97]. In conclusion, through the use of germ-free mice as a proof of concept, our previous and current studies have underscored the pivotal role of gut microbes, specifically, *B. breve* UCC2003, in the control of metabolic and cellular pathways in the placenta and in the fetal liver and fetal brain (summarized in Figure 5). While the precise mechanisms governing the alterations induced by *B. breve* UCC2003 necessitate further exploration, our study provides clear evidence that maternal oral intake of probiotics can influence fetal organogenesis.

## 4 Limitations of the study

While we have identified changes in gene and protein expression, how these relate to the structural organization of the fetal brain following maternal administration of *B. breve* UCC2003 remains unclear. Additionally, to fully understand the molecular changes induced in the fetal brain by maternal supplementation of *B. breve,* unbiased analyses like RNA-sequencing and/or phospho-proteomics are needed. As the brain is very heterogenous in nature, with functionally specialised regions under discrete developmental control, future research should employ single cell and spatial techniques to elucidate how distinct areas and cell types in the fetal brain respond to maternal *B. breve* supplementation [98]. It is noteworthy that, although samples analysed represented both fetal sexes (see Table S4), our study is under-powered to determine whether males and females varied in their response to maternal BIF treatment. This is an important area for future research, given that previous work has identified genes can be sexually-dimorphically expressed in the developing brain even prior to gonadal hormone production [99]. Additionally, the inclusion of specific-pathogen-free mice in the study, as we did previously [32], would have provided valuable information to comprehend the direction of the metabolic changes observed in the BIF group. This is important to highlight as the exposure to a microbial challenge in the GF mouse could have also resulted in elevated immune responses. In this regard, it is plausible that the metabolite levels in the BIF group could be within normal ranges for the gestational day and for fetal brain development of the specific-pathogen-free mice (rather than the metabolites being used up more in the BIF treated compared to untreated GF mice). Conversely, untreated GF fetuses might increase the abundance of these metabolites as a mechanism to preserve fetal brain development and maturation in response to growth restriction. For instance, in growth-restricted piglets, there is an increased activity of aromatic amino acid decarboxylase [100]. Additionally, the amino acid alanine, which our study found to be altered, is elevated in rats experiencing fetal growth restriction [101]. A more sensitive mass spectrometry method may also allow neurotransmitter homologues like GABA to be assessed. This is relevant as there are some data that indicate *Bifidobacterium* can produce these and would be relevant for interpreting our findings [102]. Furthermore, the lack of postnatal investigations, including behavioural studies, has constrained our ability to ascertain potential implications of *B. breve* UCC2003 on both short- and long-term neurocognitive outcomes in offspring. In this regard, previous studies have shown that the microbiota can affect host behaviour [57]. Interestingly, another *Bifidobacterium* strain, *B. longum* NCC3001, has been shown to normalize anxiety-like behaviour in a mouse model of chronic colitis by activating vagus-nerve pathways at the level of the enteric nervous system, reinforcing the concept of a gut–brain communication axis [103]. Considering the changes that we have found *in utero*, future work should explore the potential fetal programming effects on the autonomic nervous system (enteric nervous system and vagus nerve), the neuroendocrine system and the hypothalamic–pituitary–adrenal (HPA) axis. Studies are also needed to monitor the growth and behaviour of offspring into adult-life.

## 5 Materials and methods

### 5.1 Bacterial strain, growing conditions and lyophilization

*B. breve* UCC2003 generation/growing conditions were previously described [32]. Briefly, *B. breve* was grown in De Man, Rogosa and Sharpe agar (MRS) under anaerobic conditions overnight. Bacterial cell pellet was resuspended in 10% milk powder and lyophilised in 200 ml volume. Lyophilised *B. breve* was reconstituted with 500 μl PBS. Concentration of *B. breve* was 10^10^ CFU/ml and all batches were tested for contamination upon reconstitution on Luria-Bertani (LB) and Brain-Heart Infusion (BHI) plates under anaerobic and aerobic conditions at 37 °C.

### 5.2 Animal work ethics statement

All animal work was conducted at the University of East Anglia (UK) under the UK Regulation of Animals (Scientific Procedures) Act of 1986 and approved by the UK Home Office and the UEA Ethical Review Committee (project license PDADA1B0C).

### 5.3 Animal model and experimental design

The animal model employed for this study was previously described [32] and the experimental design is depicted in Figure S1. Briefly, GF mice were housed under a 12:12 h light/dark with free access to food and water. GF-C57BL/6J mice were time mated and on gestational day (GD) GD9.5, pregnant GF mice were transferred to individually ventilated cages. Treatment with the probiotic started on GD10 (+2 extra doses on GD12 and GD14) by providing 100 μL of reconstituted lyophilised *B. breve* UCC2003 or 100 μL vehicle control (PBS, 4 % skimmed milk powder) by oral gavage. The group that received the three doses of *B. breve* UCC2003 received the name of BIF group (n = 6 pregnant mice), and the other cohort of mice (treated with vehicle solution) was named as GF group (n = 5 pregnant mice). As previously described, our probiotic was formulated in a similar fashion to commercial probiotics preparations. Moreover, the time frame was selected to reflect a potential frame in which women would take probiotics once their pregnancy is confirmed. The colonisation levels of *B. breve* in pregnant mice can be found in our previous publication [32].

### 5.4 RNA extraction

Fetal brain RNA was extracted from 1 to 3 fetuses randomly selected per litter with RNeasy plus Mini Kits (Qiagen) as previously described [96]. Reverse transcription was performed using the cDNA reverse transcription kit (Applied Biosystems) following manufacturer's instructions. Samples were analysed with a StepOne real-time PCR machine (ThermoFisher) in duplicates using SYBR Green qPCR master mix (Applied Biosystems, ThermoFisher). Gene expression was normalized to the geometric mean expression of two reference genes, *Actb* and *Gapdh*. Analysis was performed using the 2-ΔΔCt method [104] and the primer sequences used can be found in Table S2.

### 5.5 Protein extraction and western blotting assays

Brain protein extraction and Western blotting were performed as previously described [96]. Briefly, brain samples (1 fetus randomly selected per litter) were lysed with radioimmunoprecipitation assay (RIPA) lysis buffer (R0278-50M, Sigma Aldrich) supplemented with protease inhibitor cocktail mix (11836170001, Roche), 1 mM β-glycerophosphate (G-9891, Sigma Aldrich) and 1 mM sodium orthovanadate (S65089891, Sigma Aldrich). After protein quantification with BCA protein assay kit (23,225, ThermoFisher), samples were mixed with SDS gel loading buffer (L-4390, Sigma Aldrich) and protein denaturalization performed at 90 °C for 5 min. After electrophoresis, membranes were blocked with 5% fetal bovine serum (A2153-100G, Sigma Aldrich) or semi-skimmed milk (Marvel) and incubated overnight with primary antibodies described in Table S3. The day after, membranes were incubated with secondary antibodies conjugated with horseradish peroxidase (HRP) (1:10,000 NA934 or NA931, Amersham) and exposed to ECL substrate (SuperSignal West Femto, ThermoFisher) for chemioluminiscence detection. Images were taken with the iBright instrument (ThermoFisher) Pixel intensity of protein bands was analysed with ImageJ software.

### 5.6 Metabolite extraction and nuclear magnetic resonance (NMR) spectroscopy

Fetal brain metabolites were extracted as described elsewhere [32]. Briefly, frozen tissue (∼14 mg) from 1 fetus randomly selected per litter was mixed with 200 μL of ice-cold methanol (Fisher Scientific) and 42.5 μL of ultra-pure cold water. Samples were vortexed and tissue was disrupted with a a tissue lyser (Qiagen) and ∼ 15–20 glass beads (Merck) for for 2 × 2 min. Subsequently, 100 μL of ice-cold chloroform (Merck) and 100 μL of ultra-pure cold water were added to the mixture. Samples were incubated on ice for 15 min and then transferred into sterile microcentrifuge tubes and centrifuged for 3 min at 17,000×*g*. The aqueous phase was transferred into new tubes and speed-vacuumed for 30 min at 50 °C and 30 min without heating prior to reconstitution with phosphate buffer solution at 600 μL. Samples were subjected to NMR spectroscopy. The 1H NMR spectra were recorded at 600 MHz on a Bruker AVANCE spectrometer (Bruker BioSpin GmbH, Germany) running Topspin 2.0 software. Fetal brain metabolites were quantified with the software Chenomx® NMR Suite 7.0™.

### 5.7 Fetal sex

Fetal sex, determined by the detection of the Sry gene, was performed retrospectively on fetal tissue incubated with lysis buffer (composed with KCl, 1M Tris-HCl, 1M MgCl2, Gelatin, Tween-20, Nonidet P-40 and Proteinase K). Then, samples were mixed with Taq Ready PCR system (Sigma), specific primers (Sry: FPrimer: 5′-GTGGGTTCCTGTCCCACTGC-3′, RPrimer: 5′-GGCCATGTCAAGCGCCCCAT-3′ and PCR autosomal gene control: FPrimer: 5′-TGGTTGGCATTTTATCCCTAGAAC-3′, RPrimer: 5′-GCAACATGGCAACTGGAAACA-3′). Samples were run in agarose gel electrophoresis.

### 5.8 Statistical analysis

All statistical analyses and sample sizes are shown in each figure legend. Only samples from viable fetuses were analysed. Statistical analysis was performed with GraphPad Prism software (GraphPad v9, San Diego, CA), SAS/STAT 9.0 (Statistical System Institute Inc. Cary, NC, USA) and Microsoft Excel (v2010). Identification and removal of outliers was performed with the ROUT method [105]. For parameters involving only one fetus per litter (Western blot data and metabolomics), a one-way ANOVA was employed with group as a fixed effect and means comparisons were made using the Fisher test (general linear model-GLM model). Conversely, parameters involving more than one fetus per litter (qPCR) were analysed using a one-way ANOVA with the group as fixed effect and each fetus treated as a repeated measure, employing the Fisher test for means comparisons (linear mixed model - MIXED model). In the MIXED model, fetuses coming from the same litter were nested. In both statistical analyses, litter size served as a covariate. The significance threshold for all statistical tests used in this study was set at *p* < 0.05. Figures in the manuscript show mean ± SEM alongside individual data points (raw data). However, the reported mean ± SEM bars have been adjusted for repeated measures and/or litter size. Data were graphed in GraphPad and figure panels were merged with Adobe Illustrator to display corrected mean ± SEM and individual dots.

## Funding

This work was supported by (JL-T) Sir Henry 10.13039/100010269Wellcome Postdoctoral Fellowship (220456/Z/20/Z), 10.13039/501100000288Newton International Fellowship from the Royal Society (NF170988/RG90199) and Attraction of Talent Grant from the Community of Madrid (grant No. 2023-T1/SAL-GL-28960, CESAR NOMBELA fellowship). L.J.H. is supported by Wellcome Trust Investigator Award 220876/Z/20/Z; the 10.13039/501100000268Biotechnology and Biological Sciences Research Council (BBSRC), Institute Strategic Programme Gut Microbes and Health BB/R012490/1, and its constituent projects BBS/E/F/000PR10353 and BBS/E/F/000PR10356, and the BBSRC Institute Strategic Programme Food Microbiome and Health BB/X011054/1 and its constituent project BBS/E/F/000PR13631. ANS-P is supported by a 10.13039/501100001255Lister Institute of Preventative Medicine Research Prize (RG93692). DvS is a member of the 10.13039/501100014745APC Microbiome Ireland research centre funded by 10.13039/501100001602Science Foundation Ireland (SFI) through the Irish Government’s National Development Plan (Grant numbers SFI/12/RC/2273a and SFI/12/RC/2273b).

## CRediT authorship contribution statement

**Jorge Lopez-Tello:** Writing – review & editing, Writing – original draft, Visualization, Validation, Supervision, Resources, Project administration, Methodology, Investigation, Funding acquisition, Formal analysis, Data curation, Conceptualization. **Raymond Kiu:** Writing – review & editing, Methodology, Investigation, Formal analysis, Data curation. **Zoe Schofield:** Writing – review & editing, Methodology, Investigation, Data curation. **Cindy X.W. Zhang:** Writing – review & editing, Investigation. **Douwe van Sinderen:** Writing – review & editing, Resources. **Gwénaëlle Le Gall:** Writing – review & editing, Methodology, Investigation, Formal analysis, Data curation. **Lindsay J. Hall:** Writing – review & editing, Writing – original draft, Supervision, Resources, Project administration, Methodology, Investigation, Funding acquisition, Conceptualization. **Amanda N. Sferruzzi-Perri:** Writing – review & editing, Writing – original draft, Supervision, Resources, Project administration, Methodology, Investigation, Funding acquisition, Conceptualization.

## Declaration of competing interest

The authors declare that they have no known competing financial interests or personal relationships that could have appeared to influence the work reported in this paper.

## Data availability

Data will be made available on request.

## Figures and Tables

**figs1:**
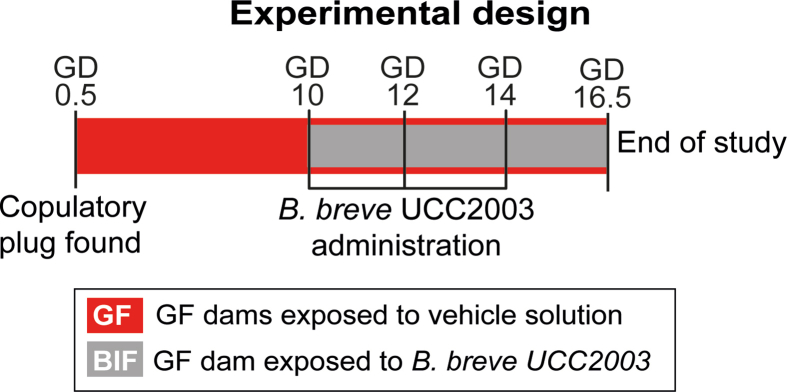


## Appendix A Supplementary data

Table S1 **List of metabolites analysed in fetal brains.** Data analysed by one-way ANOVA, with the group as fixed effect and means comparisons made by Fisher test (general linear model-GLM model). Litter size added as a covariate. Data displayed as mean ± SEM. Values were considered statistically significant with *P* < 0.05. Additional metabolites can be found in Table 1. Table S1
