# Supplementary material for: Maternal gut Bifidobacterium breve modifies fetal brain metabolism in germ-free mice
Source: Mol Metab. 2024 Aug 8;88:102004. doi: 10.1016/j.molmet.2024.102004 (PMC11401360; doi:10.1016/j.molmet.2024.102004)
Supplement: Table S1 — List of metabolites analysed in fetal brains. Data analysed by one-way ANOVA, with the group as fixed effect and means comparisons made by Fisher test (general linear model-GLM model). Litter size added as a covariate. Data displayed as mean ± SEM. Values were considered statistically significant with P < 0.05. Additional metabolites can be found in Table 1. [file mmc1.pdf]

	GF (n=5)		BIF (n=5)		
Metabolite (mmol/Kg)	GF (mean)	GF (SEM)	BIF (mean)	BIF (SEM)	P value
Betaine	0.2676	0.061381268	0.1178	0.013734628	0.0569
Lactate	10.6016	1.063430844	7.424	1.168295125	0.065
Cytidine	0.1232	0.0354336	0.04	0.014142136	0.0675
Glutamate	4.1476	0.267042618	3.1724	0.335783948	0.0705
Phenylalanine	0.2488	0.074205391	0.0832	0.021822924	0.0836
Methionine	0.2744	0.066123067	0.1298	0.020468024	0.0839
Histidine	0.1888	0.063594339	0.0514	0.0168333	0.0924
Isoleucine	0.143333333	0.025075442	0.1056	0.01319318	0.0967
Tyrosine	0.3464	0.107918766	0.1228	0.036240033	0.108
Tryptophan	0.1274	0.036243068	0.0584	0.026777976	0.1172
Inosine monophosphate	0.2144	0.078869893	0.4002	0.066516464	0.1278
Serine	1.3314	0.461185928	0.4922	0.100293769	0.1399
Glycine	1.611	0.496477425	0.83	0.127705521	0.1437
Niacinamide	0.0952	0.02623814	0.0408	0.016989408	0.1452
Asparagine	0.5632	0.175304991	0.25	0.048395248	0.1524
Fumarate	0.0936	0.023262846	0.0526	0.00762627	0.1528
Choline	0.5425	0.184209346	0.2742	0.027673814	0.1575
Glutathione	0.2504	0.046117892	0.3622	0.05051574	0.1679
Proline	0.430666667	0.042600209	0.3314	0.037349163	0.1785
Citrulline	0.4308	0.113535193	0.2496	0.043513906	0.1991
Ornithine	0.09225	0.030931039	0.0488	0.01081388	0.2133
Adenine	0.16918123	0.050174141	0.12429126	0.019631607	0.2164
Uracil	0.11425	0.070642734	0.0216	0.014661514	0.2278
Inosine	0.12891529	0.035882308	0.08908471	0.020438689	0.2414
Pyruvate	0.0496	0.010628264	0.0328	0.0069383	0.2417
Malate	0.55375	0.175559285	0.3388	0.047863765	0.2627
Taurine	14.7964	1.58817227	12.3998	1.241554807	0.2897
Pantothenate	0.0522	0.002222611	0.0432	0.007095069	0.2962
Lysine	0.3288	0.138555909	0.162	0.038836838	0.3147
Acetate	0.5278	0.113114721	0.407	0.046716164	0.3744
sn-Glycero-3-phosphocholine	0.2238	0.037650232	0.1634	0.049839342	0.3806
Glutamine	2.8718	0.63608556	2.1612	0.306117526	0.3812
NAD+	0.051	0.021286146	0.0732	0.006126989	0.3815
Creatine	4.4308	0.469284391	3.8406	0.464407214	0.4083
Orotate	0.5158	0.033486415	0.5586	0.046636466	0.4211
O-Phosphocholine	1.825	0.298927249	1.5226	0.17404011	0.4267
GABA (4-Aminobutyrate)	1.1436	0.281181898	0.8652	0.198494937	0.4329
Glycerol	0.2392871	0.044351813	0.20917032	0.029064411	0.4448
Cytidine monophosphate	0.1134	0.05004458	0.1518	0.010485228	0.4915
Malonate	0.05748544	0.012213381	0.05061165	0.007870197	0.4957
Guanosine triphosphate	0.0904	0.028748217	0.1134	0.016877796	0.5351
Guanosine monophosphate	0.792	0.324641187	1.0286	0.138220693	0.5579
Uridine monophosphate	0.2018	0.082812076	0.2548	0.022889736	0.5877
O-Acetylcarnitine	0.0338	0.009488941	0.0282	0.005851496	0.6155
UDP-N-Acetylglucosamine	0.1228	0.050792125	0.1534	0.01913792	0.6236
O-Phosphoethanolamine	2.335	0.288965915	2.1036	0.295425896	0.6258
Ascorbate	1.5478	0.57046283	1.8294	0.253491933	0.6786
N-acetylaspartylglutamate	1.3056	0.277008953	1.4528	0.225577348	0.699
Ethanolamine	0.09775	0.007824907	0.1044	0.017281782	0.7009
Creatine phosphate	0.0546	0.034152013	0.0706	0.016182089	0.7014
2-Aminobutyrate	0.0582	0.010627323	0.0662	0.018347752	0.7183
Glucose	0.245333333	0.050452398	0.2102	0.065521294	0.7184
Adenosine monophosphate	0.2926	0.11953602	0.3416	0.02691951	0.729
2-Hydroxyvalerate	0.0362	0.007059745	0.0332	0.00287054	0.7317
UDP-glucose	0.1272	0.053163333	0.1486	0.023187497	0.7501
Sucrose	0.07625	0.034424737	0.0612	0.022828491	0.7843
Ethanol	0.2118	0.039909147	0.2238	0.013078226	0.796
UDP-galactose	0.0652	0.028690417	0.0566	0.018321026	0.8261
NADP+	0.0164	0.007061161	0.015	0.004	0.8412
Pyroglutamate	0.129333333	0.026263621	0.1238	0.008974408	0.8559
N-Acetylaspartate	0.4952	0.088977188	0.5094	0.069656012	0.8786
Succinate	0.2128	0.064494496	0.223	0.034221338	0.88
myo-Inositol	5.4316	0.891766595	5.5872	1.051964229	0.8822
Uridine	0.010188147	0.024182179	0.10067112	0.008631338	0.9334
Adenosine diphosphate	0.2294	0.097748453	0.2268	0.028967913	0.9595
Guanosine	0.042	0.014804279	0.0408	0.002267157	0.9674
Methanol	0.9896	0.100898761	0.9924	0.117858644	0.9805
Formate	0.2728	0.019903266	0.2714	0.035055099	0.9882
