# Supplementary material for: Maternal gut Bifidobacterium breve modifies fetal brain metabolism in germ-free mice
Source: Mol Metab. 2024 Aug 8;88:102004. doi: 10.1016/j.molmet.2024.102004 (PMC11401360; doi:10.1016/j.molmet.2024.102004)
Supplement: Multimedia component 2 [file mmc2.docx]

**Table S2. List of primers used for qPCR.**

|  | Forward | Reverse |
| --- | --- | --- |
| *Vegf* | CTGACGATGGCCTGGAATGT | GAGGATCCTGGGGCTGTCT |
| *P53* | TATGTGCACGTACTCTACTCTCCTC | TGCTGTGACTTCTTGTAGATG |
| *Casp3* | CAGCCAACCTCAGAGAGACA | ACAGGCCCATTTGTCCCATA |
| *Bax* | TCATGAAGACAGGGGCCTTT | GTCCACGTCAGCAATCATCC |
| *Foxm1* | CGGCCTGTGAGGGTCAAA | CTGATGTTTCACTCGGGGCA |
| *Cdk1* | ACTCGGCCTCTAAGCTCCT | AGGTTACGACGGACCCTCTC |
| *Cdk2* | CGGCTCGACACTGAGACTG | TTCTTGAGGTCCTGGTGCAG |
| *Cdk4* | CTTAGCCGAGCGTAAGGCTG | CCAGGCCGCTTAGAAACTGA |
| *Ier3* | GATGGCGAACAGGAGAAAGAG | GCGCGTTTGAACACTTCTC |
| *Klf2* | CTTCCAGCCGCATCCTTC | GCAAGACCTACACCAAGAGC |
| *Egr1* | GATAACTCGTCTCCACCATCG | AGCGCCTTCAATCCTCAAG |
| *Ms4a7* | GTTCTCCCAGGAGCAGAGTG | GCCAGGGATGCTGTCCTC |
| *Mrc1* | TTCAGCTATTGGACGCGAGG | GAATCTGACACCCAGCGGAA |
| *Ccr1* | TACTCTGGAAACACAGACTCACT | ACAGCAGTCTTTTGGCATGG |
| *Plxna3* | TAACACATGCCAGGGCAAGAA | TGTCTGGCTTGGGGAATCAC |
| *Slit1* | TAGCATGCACTCACACCTGG | CTGCTCCCCGGATATGAACC |
| *Sema3f* | GGCTAAGAGACAAGAGGGCG | CCAGTTCTGTGGACACCTCG |
| *Ntn1* | TCGCCCCTTGCATCAAGATT | GAACTTCCACCAGTCCCCTG |
| *Nrcam* | CGCTGGATGTTCCTCTCGAT | AGTCCAGGAAAAGCTTGGGG |
| *Slc7a5* | CTGCTGACACCTGTGCCATC | GGCTTCTTGAATCGGAGCC |
| *Slc7a8* | CCAGTGTGTTGGCCATGATC | TGCAACCGTTACCCCATAGAA |
| *Slc38a1* | CCTTCACAAGTACCAGAGCAC | GGCCAGCTCAAATAACGATGAT |
| *Slc38a2* | TAATCTGAGCAATGCGATTGTGG | AGATGGACGGAGTATAGCGAAAA |
| *Slc38a4* | GCGGGGACAGTATTCAGGAC | GGAACTTCTGACTTTCGGCAT |
| *Slc2a1* | GCTTATGGGCTTCTCCAAACT | GGTGACACCTCTCCCACATAC |
| *Slc2a3* | GA TCGGCTCTTTCCAGTTTG | CAA TCA TGCCACCAACAGAG |
| *Slc37a4* | GCTACTCATGATGGCTGGGA | CAAACAAGGCAATGGGACCA |
| *Slc16a1* | GCCGTCCAGTAATGATCGCT | GCAAGCCCAAGACCTCCAAT |
| *Slc16a2* | GCTTCGGCTGGATAGTGGTG | CTCCGACCCATGCTGCTT |
| *Slc16a4* | GGCTGGCGGTAACAGAGTA | CGGCCTCGGACCTGAGTATT |
| *Slc16a8* | GTGCTCTTGGTGTGCATTGG | CCGAAGTCCCGGCATAGG |
| *Slc27a1* | GGCTCCTGGAGCAGGAACA | ACGGAAGTCCCAGAAACCAA |
| *Slc27a3* | GAGAACTTGCCACCGTATGC | GGCCCCTATATCTTGGTCCA |
| *Slc27a4* | GATTCTCCCTGTTGCTCCTGT | CCATTGAAGCAAACAGCAGG |
| *Slc27a6* | AACCAAGTGGTGACATCTCTGC | TCCATAAAGTAAAGCGGGTCAG |
| *Pkb* | GCCGCCTGATCAAGTTCTCC | TTCAGATGATCCATGCGGGG |
| *Prkaa1* | CTCTATGCTTTGCTGTGTGG | GGTCCTGGTGGTTTCTGTTG |
| *Mapk1* | TGCTTTCTCTCCCGCACAAA | GGCCAGAGCCTGTTCAACTT |
| *Stat5b* | GGACTCCGTCCTTGATACCG | TCCATCGTGTCTTCCAGATCG |
| *Hif1a* | TTGGCAGCGATGACACAGAA | TGCAGGATCAGCACTACTTCG |
| *Hif2a* | TGACTCTCAAAAACGGCTCTGG | GCCGACTTGAGGTTGACAGT |
| *Ascl1* | ACTTTGGAAGCAGGATGGCAG | ACCCCTGTTTGCTGAGAACAT |
| *Sv2b* | ACAGGCTCCGTTTAAAGGCTAT | AGGCTTGTGCTGGGAGTAAC |
| *Gabra1* | ATGTTCTAGCAGGGAAGCGAG | GAGGGCTGTCCATAGCTTCTTC |
| *Gabrg1* | AAACAAGACTTCGGCTTCCCC | GCCCTCCAAACACTGGTAGC |
| *Gabrg2* | CGGGCATGAATAAAATGACGCT | TTTTGGCTTGTGAAGCCTGG |
| *Ccne1* | GACACAGCTTCGGGTCTGAG | CTGGAGCGGACTGAAAGGTC |
| *Cdc42* | GGCGGAGAAGCTGAGGACA | ACCAACAGCACCATCACCAA |
| *Axin2* | GCGCTTTGATAAGGTCCTGG | TCATGTGAGCCTCCTCTCTTTT |
| *Gli1* | CAGCATGGGAACAGAAGGACT | GAAAGGGGCGAGATGGAGAG |
| *Gapdh* | GGGAAATGAGAGAGGCCCAG | GAACAGGGAGGAGCAGAGAG |
| *Actb* | GGCTGTATTCCCCTCCATCG | CCAGTTGGTAACAATGCCATGT |
