# Supplementary material for: Maternal gut Bifidobacterium breve modifies fetal brain metabolism in germ-free mice
Source: Mol Metab. 2024 Aug 8;88:102004. doi: 10.1016/j.molmet.2024.102004 (PMC11401360; doi:10.1016/j.molmet.2024.102004)
Supplement: Multimedia component 3 [file mmc3.docx]

**Table S3. List of antibodies used for western-blotting.**

| Name of antibody | Company | Product number | Dilution |
| --- | --- | --- | --- |
| PI3K-p85 alpha | Millipore | 06-195 | 1:5000 |
| PI3K-p110 alpha | Cell Signaling | 4249 | 1:1000 |
| PI3K-p110 beta | Cell Signaling | 3011 | 1:1000 |
| Total AKT | Cell Signaling | 9272 | 1:1000 |
| Total AMPK | Cell Signaling | 2532 | 1:1000 |
| Total ERK | Cell Signaling | 4695 | 1:1000 |
| Total STAT5 | Thermo Fisher Scientific | 133600 | 1:250 |
| Phospho-AKT Thr308 | Cell Signaling | 9275 | 1:1000 |
| Phospho-AMPK Thr172 | Cell Signaling | 2531 | 1:1000 |
| Phospho-ERK Thr202/204 | Cell Signaling | 4370 | 1:1000 |
| Phospho-STAT5 Tyr694 | Cell Signaling | 9351 | 1:1000 |
| Phospho-β-Catenin Ser675 | Cell Signaling | 4176T | 1:1000 |
| HIF1 alpha | Novusbio | NB100-449 | 1:1000 |
| HIF2 alpha | Novusbio | NB100-122 | 1:1000 |
| PDHK1 | Cell Signaling | 3820 | 1:1000 |
| HSP90 | Cell Signaling | 4877 | 1:1000 |
| Frizzled-7 | Abcam | Ab64636 | 1:1000 |
| HIRA | Cell Signaling | 12463 | 1:1000 |
| Actin | Cell Signaling | 3700 | 1:5000 |
| Beta catenin | Bd Biosciences | 610153 | 1:1000 |
| OxPhos cocktail | Thermofisher | 45-8099 | 1:200 |
