# Supplementary material for: Maternal gut Bifidobacterium breve modifies fetal brain metabolism in germ-free mice
Source: Mol Metab. 2024 Aug 8;88:102004. doi: 10.1016/j.molmet.2024.102004 (PMC11401360; doi:10.1016/j.molmet.2024.102004)
Supplement: Multimedia component 4 [file mmc4.docx]

**Table S4. Fetal sex distribution for the samples used for western blotting, metabolomics and gene expression.**

| **Technique** | **GF fetuses** | **BIF fetuses** |
| --- | --- | --- |
| qPCR | 9 (3 females – 6 males) | 9 (2 females – 7 males) |
| Western blotting | 5 (1 female – 4 males) | 5 (2 females – 3 males) |
| Metabolomics | 5 (3 females – 2 males) | 4 (2 females – 3 males) |
